# Supplementary material for: Treatments and Outcomes in Neuroendocrine Patients Treated with Long-Acting Somatostatin Analogues: An Italian Real-World Propensity Score-Matched Cohort Study
Source: Biomedicines. 2025 Feb 19;13(2):515. doi: 10.3390/biomedicines13020515 (PMC11852996; doi:10.3390/biomedicines13020515)
Supplement: Supplementary file 1 [file biomedicines-13-00515-s001.zip › biomedicines-3454917-supplementary.pdf]

Supplementary material

Treatments and outcomes in neuroendocrine patients treated with long-acting somatostatin analogues: an Italian Real-World Propensity Score Matched Cohort Study

N. Ranallo et al.

Table S1. Multivariable logistic regression.The analysis was used to estimate the propensity scores of the Lanreotide cohort (i.e., 1st Line SSA treatment).

Logit model						
**Parameter**	**Odds Ratio (OR)**	**SE**	**95% CI**		**Z**	**p-value**
			**Lower limit**	**Upper limit**		
**Sex [Male]**	0.878	0.186	0.579	1.332	-0.612	0.5408
**Age**	0.988	0.009	0.971	1.006	-1.313	0.1890
**Year of diagnosis**	1.180	0.040	1.106	1.262	4.925	<0.0001
**MEN1 syndrome**	0.264	0.217	0.038	1.128	-1.622	0.1048
**Primary Tumour localization**						
Lung	1.270	0.613	0.491	3.277	0.495	0.6205
Pancreas	1.667	0.611	0.818	3.462	1.393	0.1635
Gastrointestinal	0.805	0.297	0.391	1.675	-0.587	0.5572
**Tumour Grade WHO (2017-2019)**						
G1	1.364	0.524	0.645	2.926	0.806	0.4200
G2	0.781	0.284	0.382	1.598	-0.681	0.4961
G3	0.931	0.751	0.186	4.561	-0.088	0.9296
**Ki-67**						
<3%	0.456	0.207	0.185	1.108	-1.731	0.0835
35-20%	0.560	0.241	0.240	1.304	-1.350	0.1769
>20%	0.739	0.566	0.158	3.316	-0.395	0.6930
**Metastases**						
Liver	1.349	0.435	0.723	2.571	0.929	0.3529
Lung	1.360	0.676	0.508	3.649	0.619	0.5359
Lymph nodes	1.063	0.231	0.694	1.628	0.280	0.7797
Bones	1.301	0.345	0.772	2.191	0.991	0.3216
Peritoneum	1.064	0.340	0.565	1.985	0.195	0.8457
**Carcinoid syndrome**	1.552	0.374	0.970	2.498	1.825	0.0681
**Carcinoid heart**	0.666	0.428	0.182	2.335	-0.632	0.5272
**Surgery**	1.382	0.341	0.853	2.249	1.309	0.1906

Abbreviation: CI,confidential interval; SE, standard error; Z, Z statistic; MEN1, Multiple endocrine neoplasia type; 1; Ki-67,Antigen Kiel 67

Figure S1. Common support requirement assessment. Overlap in the propensity scores distribution of lanreotide and octreotide groups.

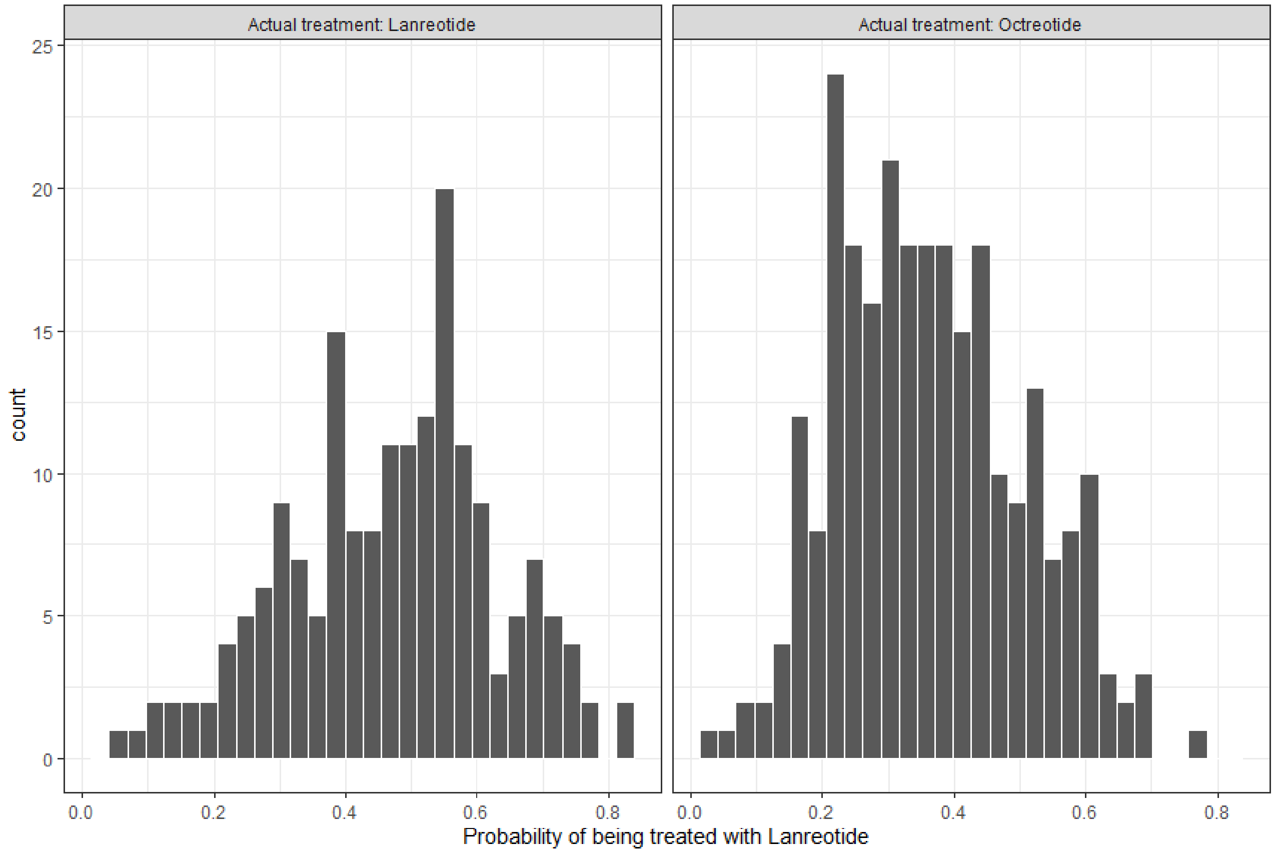

Figure S2. Distribution of propensity scores after the nearest neighbour matching. The matched treated units represent patients treated with lanreotide (N=155) in the first-line treatment, while the matched control units represent patients treated with octreotide (N=155). The unmatched (i.e. the patients who had more distant characteristics) treated (N=24) and control (N=107) units were excluded.

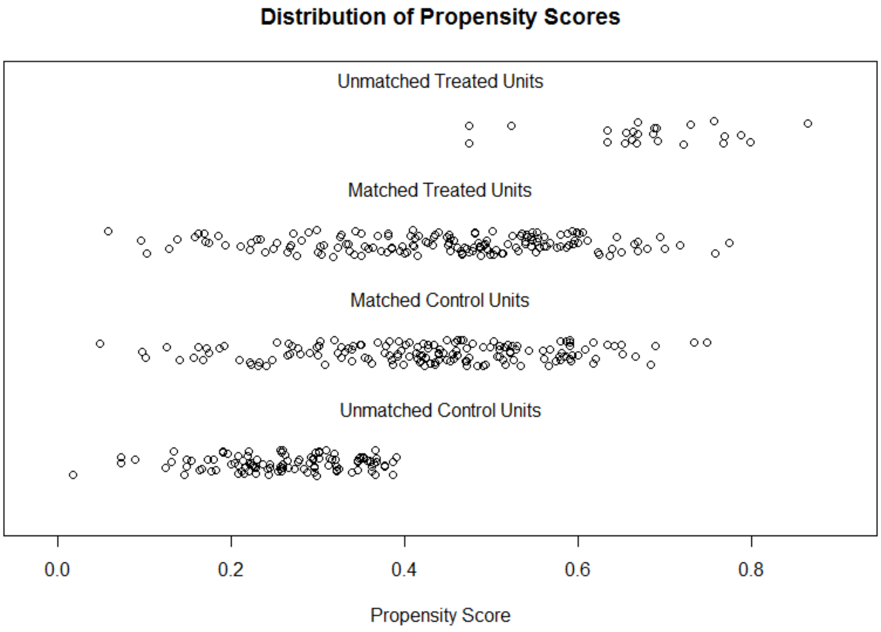

Note: Treated = Lanreotide; Control = Octreotide;

Table S2. Treatment patterns.

1st Line	2nd Line	3rd Line	4th Line	5th Line	6th Line	7th Line	N
Lanreo	Chemo	Lanreo+PRRT	Lanreo+PRRT				1
Lanreo	Chemo	PRRT					1
Lanreo	Chemo	PRRT+Chemo					1
Lanreo	Everolimus	Lanreo+PRRT	Lanreo+PRRT				1
Lanreo	Lanreo	Lanreo	Octreo	Octreo+PRRT			1
Lanreo	Lanreo	Lanreo+Everolimus	Lanreo+PRRT				1
Lanreo	Lanreo	Lanreo+PRRT					3
Lanreo	Lanreo+Chemo	Lanreo+Chemo	Lanreo+PRRT				1
Lanreo	Lanreo+Chemo	Lanreo+Everolimus	Lanreo+Chemo				1
Lanreo	Lanreo+Chemo	Lanreo+Everolimus					1
Lanreo	Lanreo+Chemo	Lanreo+PRRT	Lanreo+Sunitinib				1
Lanreo	Lanreo+Chemo	Lanreo+PRRT					1
Lanreo	Lanreo+Everolimus	Lanreo+Chemo	Lanreo+PRRT				1
Lanreo	Lanreo+Everolimus	Lanreo+Lenvatinib	Lanreo+Chemo				1
Lanreo	Lanreo+Everolimus	Lanreo+PRRT					4
Lanreo	Lanreo+Everolimus	Lanreo+Sunitinib	Lanreo+Chemo	Octreo+PRRT	Octreo+Chemo		1
Lanreo	Lanreo+PRRT	Chemo	PRRT	Everolimus			1
Lanreo	Lanreo+PRRT	Lanreo	Octreo	Octreo+Everolimus	Octreo+Chemo	Octreo+Chemo	1
Lanreo	Lanreo+PRRT	Lanreo+Chemo	Lanreo+Chemo	Chemo			1
Lanreo	Lanreo+PRRT	Lanreo+Chemo	Lanreo+PRRT				1
Lanreo	Lanreo+PRRT	Lanreo+Everolimus					2
Lanreo	Lanreo+PRRT	Lanreo+PRRT					3
Lanreo	Lanreo+PRRT	Octreo					2
Lanreo	Lanreo+PRRT						32
Lanreo	Lanreo+PRRT+Chemo	Lanreo+Everolimus					1
Lanreo	Lanreo+PRRT+Chemo						2
Lanreo	Lanreo+Sunitinib	Lanreo+Chemo	Lanreo+PRRT				1

1st Line	2nd Line	3rd Line	4th Line	5th Line	6th Line	7th Line	N
Lanreo	Lanreo+Sunitinib						1
Lanreo	Octreo	Octreo+PRRT					1
Lanreo	Octreo	Panitumumab	PRRT				1
Lanreo	Octreo+Everolimus	Octreo+PRRT	Lanreo+PRRT	Lanreo+Chemo			1
Lanreo	Octreo+PRRT	Octreo+PRRT					1
Lanreo	Octreo+PRRT						2
Lanreo	PRRT	Lanreo+Everolimus					1
Lanreo	PRRT						3
Lanreo							15
Lanreo+Chemo	Chemo						1
Lanreo+Chemo	Lanreo+Chemo	Lanreo+Chemo	Lanreo+everolimus	Lanreo+Chemo	Lanreo+Sunitinib	Lanreo+Chemo	1
Lanreo+Chemo	Lanreo+Chemo	Lanreo+PRRT					1
Lanreo+Chemo	Lanreo+Chemo	Lanreo+Sunitinib	Lanreo+Chemo	Lanreo+PRRT			1
Lanreo+Chemo	Lanreo+Chemo						2
Lanreo+Chemo	Lanreo+Everolimus	Lanreo+PRRT	Lanreo+Chemo	Lanreo+PRRT			1
Lanreo+Chemo	Lanreo+Everolimus	Lanreo+PRRT					1
Lanreo+Chemo	Lanreo+Everolimus	Lanreo+Sunitinib+PRRT					1
Lanreo+Chemo	Lanreo+PRRT	Lanreo+Everolimus	Lanreo+Chemo	Lanreo+PRRT			1
Lanreo+Chemo	Lanreo+PRRT	Lanreo+Everolimus	Lanreo+PRRT	Sunitinib			1
Lanreo+Chemo	Lanreo+PRRT	Lanreo+PRRT					1
Lanreo+Chemo	Lanreo+PRRT	Octreo+Chemo	Lanreo+Chemo	Lanreo+Everolimus	Chemo		1
Lanreo+Chemo	Lanreo+PRRT						5
Lanreo+Chemo	Lanreo+PRRT+Chemo						2
Lanreo+Chemo	PRRT+Chemo						1
Lanreo+Chemo							2
Lanreo+Everolimus	Lanreo+Chemo	Lanreo+PRRT	Lanreo+Chemo	Lanreo+PRRT			1
Lanreo+Everolimus	Lanreo+Chemo						1
Lanreo+Everolimus	Lanreo+PRRT						1
Lanreo+Everolimus	Lanreo+Sunitinib	Lanreo+PRRT	Lanreo+Chemo				1

1st Line	2nd Line	3rd Line	4th Line	5th Line	6th Line	7th Line	N
Lanreo+PRRT	PRRT						1
Lanreo+PRRT	Lanreo						1
Lanreo+PRRT	Lanreo+Chemo	Lanreo+Chemo	Lanreo+PRRT				1
Lanreo+PRRT	Lanreo+Chemo	Lanreo+Chemo					1
Lanreo+PRRT	Lanreo+PRRT	Everolimus					1
Lanreo+PRRT	Lanreo+PRRT	Lanreo+Everolimus	Lanreo+Chemo				1
Lanreo+PRRT	Lanreo+PRRT						2
Lanreo+PRRT	Lanreo+Sunitinib	Lanreo+Chemo					1
Lanreo+PRRT	Octreo	Octreo+Chemo					1
Lanreo+PRRT							16
Lanreo+PRRT+Chemo	Lanreo	Lanreo+PRRT					1
Lanreo+PRRT+Chemo	Lanreo+PRRT						1
Lanreo+PRRT+Chemo	Lanreo+Sunitinib	Lanreo+PRRT					2
Lanreo+PRRT+Chemo							1
Lanreo+Sunitinib	Lanreo+Chemo	Lanreo+PRRT					1
Lanreo+Sunitinib	Lanreo+PRRT	Lanreo+Chemo	Lanreo+Chemo	Octreo+Everolimus	Octreo+Chemo	Octreo+PRRT	1
Lanreo+Sunitinib	PRRT						1
Octreo	Chemo	Octreo+PRRT					1
Octreo	Lanreo	Lanreo+PRRT	Lanreo+Chemo				1
Octreo	Lanreo	Lanreo+PRRT	Lanreo+PRRT				1
Octreo	Lanreo	Lanreo+PRRT					2
Octreo	Lanreo						3
Octreo	Lanreo+Chemo	Chemo	everolimus				1
Octreo	Lanreo+Chemo	Lanreo+Chemo	Lanreo				1
Octreo	Lanreo+Everolimus	Lanreo+PRRT+Chemo					1
Octreo	Lanreo+Everolimus	PRRT+Chemo					1
Octreo	Lanreo+PRRT	Lanreo+Chemo	Lanreo+Chemo	Lanreo+PRRT			1
Octreo	Lanreo+PRRT	Lanreo+PRRT	Lanreo+Chemo				1
Octreo	Lanreo+PRRT	Lanreo+PRRT					1

1st Line	2nd Line	3rd Line	4th Line	5th Line	6th Line	7th Line	N
Octreo	Lanreo+PRRT						3
Octreo	Octreo	Octreo+PRRT	Lanreo				1
Octreo	Octreo	Octreo+PRRT					2
Octreo	Octreo						1
Octreo	Octreo+Chemo	Chemo	Lanreo+PRRT	Lanreo+Everolimus			1
Octreo	Octreo+Chemo	Lanreo+PRRT					1
Octreo	Octreo+Chemo	Octreo+PRRT	Octreo+Everolimus				1
Octreo	Octreo+Chemo	Octreo+PRRT					2
Octreo	Octreo+Chemo	Octreo+PRRT+Chemo					1
Octreo	Octreo+Everolimus	Octreo+Chemo	PRRT				1
Octreo	Octreo+Everolimus	Octreo+PRRT	Lanreo				1
Octreo	Octreo+Everolimus	Octreo+PRRT+Chemo					1
Octreo	Octreo+PRRT	Lanreo					2
Octreo	Octreo+PRRT	Lanreo+Chemo					1
Octreo	Octreo+PRRT	Lanreo+Everolimus	Lanreo	Lanreo+Chemo			1
Octreo	Octreo+PRRT	Lanreo+Everolimus					1
Octreo	Octreo+PRRT	Lanreo+PRRT					3
Octreo	Octreo+PRRT	Octreo+Everolimus	Octreo+PRRT	Octreo+Chemo			1
Octreo	Octreo+PRRT	Octreo+Everolimus					2
Octreo	Octreo+PRRT	Octreo+PRRT					2
Octreo	Octreo+PRRT						35
Octreo	Octreo+PRRT+Chemo	Octreo+Chemo	Octreo+Everolimus				1
Octreo	Octreo+PRRT+Chemo						2
Octreo	PRRT	Everolimus	Chemo	PRRT	Lanreo+Chemo		1
Octreo	PRRT	Everolimus	Chemo				1
Octreo	PRRT	Octreo	Octreo+Everolimus				1
Octreo	PRRT	Octreo+Chemo	Lanreo	Lanreo+PRRT			1
Octreo	PRRT	Octreo+Chemo	Lanreo				1
Octreo	PRRT						7

1st Line	2nd Line	3rd Line	4th Line	5th Line	6th Line	7th Line	N
Octreo							8
Octreo+							1
Octreo+Chemo	Chemo	Octreo+PRRT					1
Octreo+Chemo	Lanreo+PRRT						1
Octreo+Chemo	Lanreo+PRRT+Chemo	Lanreo+PRRT					1
Octreo+Chemo	Octreo+Chemo	Octreo+PRRT					1
Octreo+Chemo	Octreo+everolimus	Octreo+PRRT					1
Octreo+Chemo	Octreo+PRRT	Lanreo+Chemo	Lanreo+Chemo	Lanreo+Chemo			1
Octreo+Chemo	Octreo+PRRT	Lanreo+ Sunitinib	Lanreo+Chemo	Lanreo+Chemo	Lanreo+Everolimus	Lanreo+PRRT	1
Octreo+Chemo	Octreo+PRRT	PRRT					1
Octreo+Chemo	Octreo+PRRT						2
Octreo+Chemo	Octreo+PRRT+Chemo						3
Octreo+everolimus	Lanreo						1
Octreo+everolimus	Octreo+Chemo	Octreo+Chemo	Lanreo+PRRT				1
Octreo+everolimus	Octreo+PRRT	Lanreo					1
Octreo+everolimus	Octreo+PRRT	Lanreo+Chemo	Lanreo+Chemo				1
Octreo+everolimus	Octreo+PRRT	Octreo+sunitinib	Octreo+PRRT				1
Octreo+everolimus	Octreo+PRRT						1
Octreo+everolimus	Octreo+Sunitinib	Octreo+PRRT					1
Octreo+everolimus	PRRT+Chemo						1
Octreo+PRRT	Lanreo	Lanreo+PRRT					1
Octreo+PRRT	Lanreo						1
Octreo+PRRT	Octreo+Chemo	Lanreo	Lanreo+PRRT				1
Octreo+PRRT	Octreo+Chemo	Octreo+Chemo	Octreo+Everolimus	Octreo+Chemo	Octreo+PRRT		1
Octreo+PRRT	Octreo+PRRT	Octreo					1
Octreo+PRRT	Octreo+PRRT						1
Octreo+PRRT							19
Octreo+PRRT+Chemo							4
Octreo+Target+Chemo	Octreo+PRRT	Octreo+Everolimus	Lanreo+PRRT	Lanreo+Chemo			1

1st Line	2nd Line	3rd Line	4th Line	5th Line	6th Line	7th Line	N
Octreo+Target+Chemo	Octreo+PRRT						1

Table S3. Treatment patterns for lung primary tumour localization patients.

1st Line	2nd Line	3rd Line	4th Line	5th Line	6th Line	7th Line	N
Lanreo	Lanreo+Everolimus	Lanreo+PRRT					1
Lanreo	Lanreo+PRRT	Lanreo	Octreo	Octreo+Everolimus	Octreo+Chemo	Octreo+Chemo	1
Lanreo	Lanreo+PRRT	Lanreo+Chemo	Lanreo+Chemo	Chemo			1
Lanreo	Lanreo+PRRT	Lanreo+Everolimus					1
Lanreo	Lanreo+PRRT						1
Lanreo	PRRT						1
Lanreo							2
Lanreo+Chemo	Chemo						1
Lanreo+Chemo	Lanreo+PRRT	Octreo+Chemo	Lanreo+Chemo	Lanreo+Everolimus	Chemo		1
Lanreo+PRRT							2
Octreo	Lanreo	Lanreo+PRRT	Lanreo+Chemo				1
Octreo	Lanreo+Chemo	Chemo	everolimus				1
Octreo	Lanreo+Chemo	Lanreo+Chemo	Lanreo				1
Octreo	Octreo+PRRT	Octreo+PRRT					1
Octreo	Octreo+PRRT						1
Octreo	PRRT	Everolimus	Chemo	PRRT	Lanreo+Chemo		1
Octreo	PRRT	Octreo+Chemo	Lanreo				1
Octreo	PRRT						3
Octreo							1
Octreo+PRRT							2

Table S4. Treatment patterns for pancreatic primary tumour localization patients.

1st Line	2nd Line	3rd Line	4th Line	5th Line	6th Line	7th Line	N
Lanreo	Chemo	PRRT					1
Lanreo	Chemo	PRRT+Chemo					1
Lanreo	Everolimus	Lanreo+PRRT	Lanreo+PRRT				1
Lanreo	Lanreo	Lanreo+PRRT					2
Lanreo	Lanreo+Chemo	Lanreo+Everolimus	Lanreo+Chemo				1
Lanreo	Lanreo+Chemo	Lanreo+PRRT	Lanreo+Sunitinib				1
Lanreo	Lanreo+Everolimus	Lanreo+Chemo	Lanreo+PRRT				1
Lanreo	Lanreo+Everolimus	Lanreo+Lenvatinib	Lanreo+Chemo				1
Lanreo	Lanreo+Everolimus	Lanreo+PRRT					3
Lanreo	Lanreo+Everolimus	Lanreo+Sunitinib	Lanreo+Chemo	Octreo+PRRT	Octreo+Chemo		1
Lanreo	Lanreo+PRRT	Chemo	PRRT	Everolimus			1
Lanreo	Lanreo+PRRT						10
Lanreo	Lanreo+ Sunitinib	Lanreo+Chemo	Lanreo+PRRT				1
Lanreo	Lanreo+ Sunitinib						1
Lanreo	Octreo+Everolimus	Octreo+PRRT	Lanreo+PRRT	Lanreo+Chemo			1
Lanreo							5
Lanreo+Chemo	Lanreo+Chemo	Lanreo+Chemo	Lanreo+everolimus	Lanreo+Chemo	Lanreo+Sunitinib	Lanreo+Chemo	1
Lanreo+Chemo	Lanreo+Chemo	Lanreo+PRRT					1
Lanreo+Chemo	Lanreo+Chemo	Lanreo+ Sunitinib	Lanreo+Chemo	Lanreo+PRRT			1
Lanreo+Chemo	Lanreo+Chemo						2
Lanreo+Chemo	Lanreo+Everolimus	Lanreo+PRRT	Lanreo+Chemo	Lanreo+PRRT			1
Lanreo+Chemo	Lanreo+Everolimus	Lanreo+PRRT					1
Lanreo+Chemo	Lanreo+Everolimus	Lanreo+Sunitinib+PRRT					1
Lanreo+Chemo	Lanreo+PRRT	Lanreo+Everolimus	Lanreo+PRRT	Sunitinib			1
Lanreo+Chemo	Lanreo+PRRT	Lanreo+PRRT					1
Lanreo+Chemo	Lanreo+PRRT						4
Lanreo+Chemo	Lanreo+PRRT+Chemo						1

1st Line	2nd Line	3rd Line	4th Line	5th Line	6th Line	7th Line	N
Lanreo+Chemo	PRRT+Chemo						1
Lanreo+everolimus	Lanreo+Chemo						1
Lanreo+everolimus	Lanreo+PRRT						1
Lanreo+everolimus	Lanreo+Sunitinib	Lanreo+PRRT	Lanreo+Chemo				1
Lanreo+PRRT	PRRT						1
Lanreo+PRRT	Lanreo+ Sunitinib	Lanreo+Chemo					1
Lanreo+PRRT							6
Lanreo+PRRT+Chemo	Lanreo	Lanreo+PRRT					1
Lanreo+PRRT+Chemo	Lanreo+PRRT						1
Lanreo+PRRT+Chemo	Lanreo+Sunitinib	Lanreo+PRRT					2
Lanreo+PRRT+Chemo							1
Lanreo+Sunitinib	Lanreo+Chemo	Lanreo+PRRT					1
Lanreo+Sunitinib	Lanreo+PRRT	Lanreo+Chemo	Lanreo+Chemo	Octreo+Everolimus	Octreo+Chemo	Octreo+PRRT	1
Lanreo+Sunitinib	PRRT						1
Octreo	Lanreo						2
Octreo	Lanreo+Everolimus	Lanreo+PRRT+Chemo					1
Octreo	Lanreo+Everolimus	PRRT+Chemo					1
Octreo	Lanreo+PRRT	Lanreo+Chemo	Lanreo+Chemo	Lanreo+PRRT			1
Octreo	Lanreo+PRRT						1
Octreo	Octreo+Chemo	Octreo+PRRT	Octreo+Everolimus				1
Octreo	Octreo+Chemo	Octreo+PRRT+Chemo					1
Octreo	Octreo+Everolimus	Octreo+Chemo	PRRT				1
Octreo	Octreo+Everolimus	Octreo+PRRT	Lanreo				1
Octreo	Octreo+Everolimus	Octreo+PRRT+Chemo					1
Octreo	Octreo+PRRT	Lanreo+Everolimus	Lanreo	Lanreo+Chemo			1
Octreo	Octreo+PRRT	Lanreo+PRRT					1
Octreo	Octreo+PRRT	Octreo+Everolimus	Octreo+PRRT	Octreo+Chemo			1
Octreo	Octreo+PRRT						10
Octreo	Octreo+PRRT+Chemo						1

1st Line	2nd Line	3rd Line	4th Line	5th Line	6th Line	7th Line	N
Octreo	PRRT	Octreo	Octreo+Everolimus				1
Octreo	PRRT						1
Octreo							1
Octreo+Chemo	Octreo+everolimus	Octreo+PRRT					1
Octreo+Chemo	Octreo+PRRT	Lanreo+Chemo	Lanreo+Chemo	Lanreo+Chemo			1
Octreo+Chemo	Octreo+PRRT	Lanreo+Sunitinib	Lanreo+Chemo	Lanreo+Chemo	Lanreo+Everolimus	Lanreo+PRRT	1
Octreo+Chemo	Octreo+PRRT	PRRT					1
Octreo+Chemo	Octreo+PRRT+Chemo						3
Octreo+everolimus	Lanreo						1
Octreo+Everolimus	Octreo+Chemo	Octreo+Chemo	Lanreo+PRRT				1
Octreo+Everolimus	Octreo+PRRT	Lanreo					1
Octreo+Everolimus	Octreo+PRRT	Lanreo+Chemo	Lanreo+Chemo				1
Octreo+Everolimus	Octreo+PRRT	Octreo+Sunitinib	Octreo+PRRT				1
Octreo+Everolimus	Octreo+PRRT						1
Octreo+Everolimus	Octreo+Sunitinib	Octreo+PRRT					1
Octreo+Everolimus	PRRT+Chemo						1
Octreo+PRRT	Lanreo						1
Octreo+PRRT	Octreo+Chemo	Lanreo	Lanreo+PRRT				1
Octreo+PRRT	Octreo+PRRT	Octreo					1
Octreo+PRRT							9
Octreo+PRRT+Chemo							3

Table S5. Treatment patterns for gastro-intestinal primary tumour localization patients.

1st Line	2nd Line	3rd Line	4th Line	5th Line	6th Line	7th Line	N
Lanreo	Chemo	Lanreo+PRRT	Lanreo+PRRT				1
Lanreo	Lanreo	Lanreo+PRRT					1
Lanreo	Lanreo+Chemo	Lanreo+PRRT					1
Lanreo	Lanreo+PRRT	Lanreo+Chemo	Lanreo+PRRT				1
Lanreo	Lanreo+PRRT	Lanreo+Everolimus					1
Lanreo	Lanreo+PRRT	Lanreo+PRRT					3
Lanreo	Lanreo+PRRT	Octreo					2
Lanreo	Lanreo+PRRT						17
Lanreo	Lanreo+PRRT+Chemo	Lanreo+Everolimus					1
Lanreo	Lanreo+PRRT+Chemo						2
Lanreo	Octreo	Octreo+PRRT					1
Lanreo	Octreo	Panitumumab	PRRT				1
Lanreo	Octreo+PRRT	Octreo+PRRT					1
Lanreo	Octreo+PRRT						2
Lanreo	PRRT	Lanreo+Everolimus					1
Lanreo	PRRT						1
Lanreo							7
Lanreo+Chemo	Lanreo+PRRT	Lanreo+Everolimus	Lanreo+Chemo	Lanreo+PRRT			1
Lanreo+Chemo	Lanreo+PRRT+Chemo						1
Lanreo+Chemo							2
Lanreo+PRRT	Lanreo						1
Lanreo+PRRT	Lanreo+Chemo	Lanreo+Chemo	Lanreo+PRRT				1
Lanreo+PRRT	Lanreo+Chemo	Lanreo+Chemo					1
Lanreo+PRRT	Lanreo+PRRT	Everolimus					1
Lanreo+PRRT	Lanreo+PRRT	Lanreo+Everolimus	Lanreo+Chemo				1
Lanreo+PRRT	Lanreo+PRRT						2

1st Line	2nd Line	3rd Line	4th Line	5th Line	6th Line	7th Line	N
Lanreo+PRRT	Octreo	Octreo+Chemo					1
Lanreo+PRRT							4
Octreo	Chemo	Octreo+PRRT					1
Octreo	Lanreo	Lanreo+PRRT	Lanreo+PRRT				1
Octreo	Lanreo	Lanreo+PRRT					2
Octreo	Lanreo						1
Octreo	Lanreo+PRRT	Lanreo+PRRT	Lanreo+Chemo				1
Octreo	Lanreo+PRRT	Lanreo+PRRT					1
Octreo	Lanreo+PRRT						2
Octreo	Octreo	Octreo+PRRT	Lanreo				1
Octreo	Octreo	Octreo+PRRT					1
Octreo	Octreo						1
Octreo	Octreo+Chemo	Chemo	Lanreo+PRRT	Lanreo+Everolimus			1
Octreo	Octreo+Chemo	Octreo+PRRT					2
Octreo	Octreo+PRRT	Lanreo					2
Octreo	Octreo+PRRT	Lanreo+Chemo					1
Octreo	Octreo+PRRT	Octreo+Everolimus					2
Octreo	Octreo+PRRT	Octreo+PRRT					1
Octreo	Octreo+PRRT						19
Octreo	Octreo+PRRT+Chemo	Octreo+Chemo	Octreo+Everolimus				1
Octreo	Octreo+PRRT+Chemo						1
Octreo	PRRT	Everolimus	Chemo				1
Octreo	PRRT	Octreo+Chemo	Lanreo	Lanreo+PRRT			1
Octreo	PRRT						2
Octreo							6
Octreo+Chemo	Lanreo+PRRT						1
Octreo+Chemo	Lanreo+PRRT+Chemo	Lanreo+PRRT					1
Octreo+Chemo	Octreo+PRRT						1
Octreo+PRRT	Lanreo	Lanreo+PRRT					1

1st Line	2nd Line	3rd Line	4th Line	5th Line	6th Line	7th Line	N
Octreo+PRRT	Octreo+Chemo	Octreo+Chemo	Octreo+ Everolimus	Octreo+Chemo	Octreo+PRRT		1
Octreo+PRRT							7
Octreo+PRRT+Chemo							1
Octreo+Target+Chemo	Octreo+PRRT	Octreo+Everolimus	Lanreo+PRRT	Lanreo+Chemo			1
Octreo+Target+Chemo	Octreo+PRRT						1

Table S6. Treatment patterns for other/unknown primary tumour localization patients.

1st Line	2nd Line	3rd Line	4th Line	5th Line	6th Line	7th Line	N
Lanreo	Lanreo	Lanreo	Octreo	Octreo+PRRT			1
Lanreo	Lanreo	Lanreo+Everolimus	Lanreo+PRRT				1
Lanreo	Lanreo+Chemo	Lanreo+Chemo	Lanreo+PRRT				1
Lanreo	Lanreo+Chemo	Lanreo+Everolimus					1
Lanreo	Lanreo+PRRT						4
Lanreo	PRRT						1
Lanreo							1
Lanreo+Chemo	Lanreo+PRRT						1
Lanreo+Everolimus	Lanreo+Chemo	Lanreo+PRRT	Lanreo+Chemo	Lanreo+PRRT			1
Lanreo+PRRT							4
Octreo	Octreo	Octreo+PRRT					1
Octreo	Octreo+Chemo	Lanreo+PRRT					1
Octreo	Octreo+PRRT	Lanreo+Everolimus					1
Octreo	Octreo+PRRT	Lanreo+PRRT					2
Octreo	Octreo+PRRT						5
Octreo	PRRT						1
Octreo							1
Octreo+Chemo	Chemo	Octreo+PRRT					1
Octreo+Chemo	Octreo+Chemo	Octreo+PRRT					1
Octreo+Chemo	Octreo+PRRT						1
Octreo+PRRT	Octreo+PRRT						1
Octreo+PRRT							1

Figure S3. Kaplan-Meier by first-line SSA treatment and primary tumour site. KM estimate of OS according to first-line SSA treatment (O= octreotide; L= Lanreotide) and primary tumour site (G.I.=Gastrointestinal tract, lung, other/unknown, pancreas).

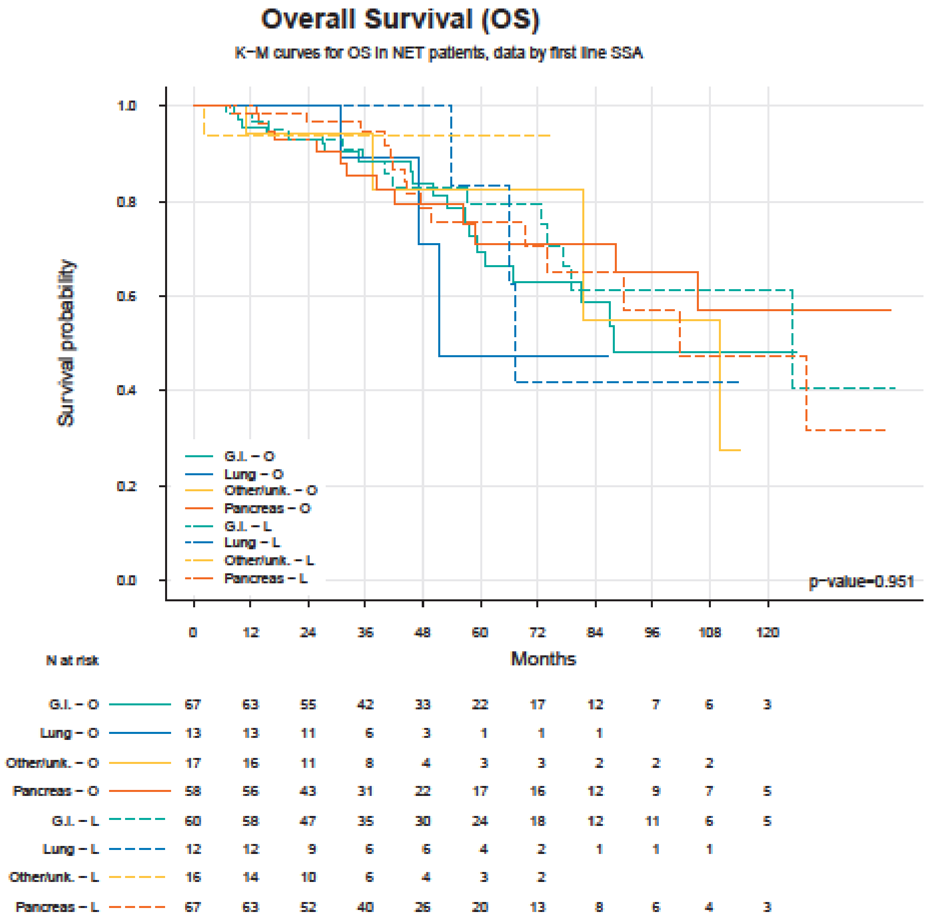

Table S7. 5-year survival estimates by 1st line SSA treatment

1st Line SSA treatment	5-year Survival	S.E.	95% C.I.
Octreotide	**69.1%**	**0.051**	**59.9% - 79.8%**
Lanreotide	**78.6%**	**0.043**	**70.5% - 87.6%**

SE, standard error;

Table S8. 5-year survival estimates by primary tumour localization

1st Line SSA treatment	5-year Survival	S.E.	95% C.I.
Gastrointestinal Tract .	**74.2%**	**0.049**	**65.3% - 84.4%**
Pancreas	**73.1%**	**0.053**	**63.5% - 84.3%**
Lung	**66.5%**	**0.140**	**44.0% - 100.0%**
Other / Unknown	**87.1%**	**0.075**	**73.5% - 100.0%**

SE, standard error;
